# Introduction to Antibody-Drug Conjugates

**DOI:** 10.3390/antib10040042

**Published:** 2021-10-27

**Authors:** Mark C. Pettinato

**Affiliations:** Department of Biomedical Engineering, School of Engineering, Catholic University of America, 620 Michigan Avenue NE, Washington, DC 20064-0001, USA; pettinato@cua.edu

**Keywords:** ADCs, monoclonal antibody, cytotoxic payload, linkers, cancer

## Abstract

Antibody-drug conjugates (ADCs) are innovative biopharmaceutical products in which a monoclonal antibody is linked to a small molecule drug with a stable linker. Most of the ADCs developed so far are for treating cancer, but there is enormous potential for using ADCs to treat other diseases. Currently, ten ADCs have been approved by the United States Food and Drug Administration (FDA), and more than 90 ADCs are under worldwide clinical development. Monoclonal antibodies have evolved from research tools to powerful therapeutics in the past 30 years. Tremendous strides have been made in antibody discovery, protein bioengineering, formulation, and delivery devices. This manuscript provides an overview of the biology, chemistry, and biophysical properties of each component of ADC design. This review summarizes the advances and challenges in the field to date, with an emphasis on antibody conjugation, linker-payload chemistry, novel payload classes, drug-antibody ratio (DAR), and product development. The review emphasizes the lessons learned in the development of oncology antibody conjugates and look towards future innovations enabling other therapeutic indications. The review discusses resistance mechanisms to ADCs, and give an opinion on future perspectives.

## 1. Introduction

Antibody-drug conjugates (ADCs) are a class of drugs designed as a targeted therapy for treating disease, but at the moment are widely used for the management or treatment of cancer [1]. They are complex molecules consisting of an antibody linked to a biologically active cytotoxic payload or drug [2].

Chemotherapy is a therapeutic option for cancer treatment. Chemotherapy, with its poor specificity towards tumor cells/tissues, is often associated with a poor therapeutic response [3] and substantial toxicities to normal healthy tissues. Unlike chemotherapy, antibody-drug conjugates target and kill tumor cells without harming the healthy cells [4], by integrating the antigen specificity of monoclonal antibodies (mAbs) with antibody fragments [5,6,7,8]. 

The concept that monoclonal antibodies directed toward targets on the surface of malignant cells could be used for drug delivery is not a recent idea. In 1913, Paul Ehrlich, a German Nobel Prize winning physicist and scientist, was the first to suggest this hypothesis, utilizing directed transport of chemo toxic agents against microbial infections or neoplasms. It took close to forty years to bring about a productive outcome of antibody drug conjugates in clinical research trials. In 1983, the first successful clinical trial utilized an anti-carcinoembryonic antigen antibody-vindesine conjugate [9]. Patient specific antibody drug conjugates increased therapeutic value by decreasing chemotoxicity, restricting the systemic circulation of destructive agents without restricting their activity on the tumor tissue. This allows clinicians to treat patients that would not tolerate systemic chemotherapy because of its toxic side effects [10].

ADCs are widely used for the management or cure of cancer, but attempts are being made to expand the use of ADCs to different diseases such as atherosclerosis, bacteremia, and inflammatory diseases, and research in these avenues is ongoing [11,12].

## 2. The Immune System

The immune system is composed of organs, cells and chemicals that attack microbial infection [13]. Antibodies have three main functions:

(1) Neutralization: Antibodies are produced in the body, binding to and inactivating foreign particles such as pathogens and toxins [14,15]. (2) Complement Recruitment System Activation: Antibodies activate the complement system destroying bacterial cells by lysis in the cellular membrane [16,17]. (3) Opsonization: Antibodies promote destruction of foreign substances by phagocytic cells [18,19]. The Immune system is a complex net- work of cells and proteins that defend the human body against infection. The immune system keeps a record of every microbe it has ever encountered, in memory cells or types of white blood cells known as B & T-lymphocytes that can make one ill [20].

The Immunological Complement System is a complex system for more than 30 pro- teins that act in unison to help destroy infectious microorganisms. Specifically, the com- plement system causes the lysis of foreign infectious cells, the phagocytosis of alien particles and cell debris and inflammation of adjacent tissue. The Complement system is an enzyme cascade, a series of protein molecules which activate each other in a sequence, defending against infection. Many complement proteins occur in serums as inactive enzyme precursors as inactive enzyme precursors proteins or zymogens; others on the cell surface [21].

### 2.1. Antibodies

Antibodies or immunoglobulins are large Y shaped proteins utilized by the immune system to recognize and eliminate foreign proteins such as pathogenic bacteria and viruses [8,9,10,13].

There are three types of antibodies currently under investigation in laboratory and clinical settings:

1. Monoclonal antibodies (mAbs),
2. Bispecific Antibodies, and
3. Antibody Drug Conjugates (ADCs).

### 2.2. Monoclonal Antibodies (mAbs)

Antibodies are glycoproteins generated by the body in reaction to a foreign molecule in the body. A monoclonal antibody (mAb) is an antibody made by cloning a specific white blood cell or unique parent cell. Monoclonal antibodies possess monovalent affinity, binding only to a molecular region or part of an antigen (epitope or antigenic determinant) that is recognized by the antibody. Polyclonal antibodies bind to various epitopes produced by several different antibody cell lineages. Higher animals have five categories of immunoglobulins, known as IgG, IgM, IgA, IgE, and IgD [14,15]. IgG is the most prevalent of these immunoglobulins. Each IgG molecule involves four polypeptide chains, two substantial chains of 50 kDa, and two minimal chains of 25 kDa, held in unison by four disulfide bridges [16,17].

Common applications of monoclonal antibodies include investigative tools for antigen, cell, and pathogen identification, column chromatography and purification methodologies, or diagnostic reagents [18].

### 2.3. Bispecific Antibodies

Bispecific monoclonal mAbs can be bioengineered, increasing the therapeutic payload targets of one mAb to two epitopes [19]. Bispecific antibodies are produced by combining a targeting forefront region of two different antibodies and recombining them to generate a product that can bind to a pair of contrasting targets [20,21]. BiTEs are one example of Bispecific antibodies that target both neoplasms and T-cells or immune cells. These BiTEs operate by inducing T cells into close quarters with neoplasms to facilitate their eradication. Due to their ability to target immune cells directly, BiTEs are deemed active immunotherapy [22,23].

### 2.4. Antibody Drug Conjugates (ADCs)

Antibody-drug conjugates (ADC) are antibodies (mAbs) engineered to utilize the capability of monoclonal antibodies by combining them to cytotoxic agents. An ideal ADC has the following features:

- A monoclonal antibody (mAb) that targets a specific cancer antigen while not harming healthy cells.
- A potent cytotoxic small molecular agent with high systemic toxicity designed to induce target cell death after being internalized in the tumor cell and discharged.
- A linker stable in circulation which releases the medicinal preparation in neoplasms.
- Monoclonal antibodies covalently linked to small molecular cytotoxic preparations that focus on the specific cancer cell to reduce total systemic toxicity.
- Strengthens the cytotoxic potential of mAbs.
- Induces higher tumor selectivity while improving the tolerability of the drug.
- As opposed to standard chemotherapeutic biologics or drugs, there is limited systemic exposure [24].

So far, there are ten ADCs [25,26,27,28,29,30,31,32,33,34] approved by the FDA, and these are listed in Table 1.

The success of ADC involves knowledge of target antigen selection, conjugate internalization by neoplasms, medicinal effectiveness, and the reliability of the linker amid medical preparation and antibody. Highly potent drug conjugation techniques, the drug-to-antibody ratio (DAR), the ramifications of conjugation on antibody characteristics, and the design of linkers are crucial in developing effective and safe ADCs [35].

## 3. Linker Technologies

In regulating pharmacokinetic characteristics, therapeutic classifications, selectivity and the comprehensive achievement of the ADC, linker technologies are instrumental [9,18]. Table 2 represents some widely used linker technologies used during in ADC development. Linkers direct the cytotoxic preparation release instrument of an ADC (non-cleavable vs. cleavable) and they also assist significantly in various ADC biochemical features.

### 3.1. Current Linker Technologies

In the United States, FDA-approved antibody–drug conjugates utilize two methodologies to bind anticancer agents including (a) cleavable linkers (a peptide linker split by cathepsin B, and (b) non-cleavable linkers (a non-cleavable thioether linker that dispenses the medical preparation after the monoclonal antibody has dissipated) [36].

### 3.2. Non-Cleavable Linkers

Non-cleavable linkers are a significant building block of antibody-drug conjugates. They disengage their cytotoxic payload during the lysosomal degradation of the antibody-drug conjugate inside the tumor environment, bypassing the non-specific dispersion of the toxic preparation [37].

Non-cleavable linkers are an innovational array of first-generation linkers with more advanced plasma stability than many cleavable linkers. Non-cleavable linkers do not seem to contain a definable payload dispersion mode, and ADCs developed with this mechanism depend on the lysosomal proteolytic decomposition of the antibody after ingestion to deliver the cytotoxic medicine. Via this strategy, the non-cleavable linker transporting the medication is attached to the conjugation amino acid within the antibody. ADCs containing non-cleavable linkers are more contingent on the membrane biology of the neoplasm than cleavable linkers. ADCs designed with non-cleavable linkers have amplified plasma stability via an enhanced therapeutic index [37]. Due to the bystander sequence, this opposition to extracellular cleavage improves the specificity of payload delivery. Numerous clinical data and in vivo studies prove ADCs containing non-cleavable linkers surpass the efficacy of those with cleavable linkers [38].

Another advantage of non-cleavable linkers is heightened plasma stability. In vivo research studies have demonstrated that non-cleavable linked ADCs outperform cleavable equivalents. mAb degradation contained in the lysosome after ADC ingestion is necessary for non-cleavable linkers to liberate their medical preparation. The payload derivative from non-cleavable ADCs destroys the target cells, and non-cleavable linkers may theoretically offer a superior medicinal opportunity versus cleavable linkers. With a reduced off-target toxicity compared to the cleavable ADCs, non-cleavable ADCs may offer improved stability and tolerability [39].

### 3.3. Cleavable Linkers

Cleavable linkers play a critical part in the design of ADCs. They are safe in blood circulation for an extensive time interval and release their cytotoxic preparations in the neoplasm microenvironment for removal of the tumor cells [40]. The three types of enzyme cleavable linkers are:

- Chemically labile linkers,
- Acid-cleavable linkers, and
- Reducible linkers.

(a) Chemically Labile Linkers

Chemically labile linkers utilized in ADCs are able to undergo fracture, augmenting the acidity of the endosomal–lysosomal route along with absorption of glutathione within cells; examples include hydrazones and disulfides [41].

(b) Acid-Cleavable Linkers

Hydrazones are examples of acid-cleavable linkers. They undergo hydrolysis and liberate the cytotoxic preparation within the acidic microenvironment of the cellular array while being precisely engineered to operate at a pH of 7 in the blood circulation while maintaining stability [42]. During clinical research trials, acid-cleavable linkers have been connected with the non-specific release of their cytotoxic medication [43].

(c) Reducible Linkers

Disulfides or reducible linkers rely on the divergence in reduction potential in the intracellular array versus plasma [44]. Reduced glutathione expressed in the tumor cytoplasm is greater than in normal cytoplasm. Additionally, tumor cells express enzymes of the protein disulfide isomerase family, influencing the reduction of the disulfide bond in the cellular environment [45]. Disulfide bond linkers maintain conjugates undamaged throughout cardiovascular circulation and are carefully bound by the glutathione abundant in high concentrations, dispensing the cytotoxic payload at the neoplasm cells.

### 3.4. Enzyme Cleavable Linkers

Enzyme cleavable linkers are chemically-cleavable linkers relying on the presence of hydrolytic enzymes in the cell. These linkers nay be peptide based or a beta-glucuronide linker [46]. The distinctive components of these enzymes amid the blood and lysosomal sections assure a well-bioengineered ADC endures binding only in the lysosomal microenvironment [47].

The two type of Enzyme Cleavable Linkers are:

- Peptide-based linkers, and
- β-Glucuronide linkers.

#### 3.4.1. Peptide-Based Linkers

Peptide-based linkers are designed to maintain ADCs integral in cardiovascular circulation, while permitting smooth dispensation of the cytotoxic medicines upon binding with specific intracellular proteases, for instance, cathepsin B [48].

Peptide linkers demonstrate superior systemic cohesion with quick enzymatic delivery of the contents in the target location, such as is the case with the valine-citrulline (Val-Cit) dipeptide linker and the phenylalanine-lysine (Phe-Lys) dipeptide linkers, as a result of inferior pH status and serum protease inhibitors [49]. Bound dipeptide linkers, i.e., Val-Ala and Val-Cit, benefit from the ADCs’ targeting design that encompasses the consecutive cleavage of the ADCs to the cognate antigen on the membrane of the target neoplasm, and the ingestion of the ADC-antigen labyrinthine throughout the endosomal–lysosomal passageway [50].

#### 3.4.2. β-Glucuronide Linkers

The β-glucuronide linker utilized in antibody-drug conjugates relies on cleavage by the enzyme β-glucuronidase, which is over expressed in the lysosome of many tumor cells. These linkers possess hydrophilic properties, allowing them to promote the solubility of the ADC when compared to dipeptide-based ADCs [51,52].

β-glucuronide linkers reduce the accumulation of hydrophobic medicines by promoting the solubility of the beta-glucuronide antibody-drug conjugates versus dipeptide-based ADCs relying on their hydrophilic properties [53,54].

## 4. Cytotoxic Payload

Usage of monoclonal antibodies (mAbs) as therapeutic agents for the management or cure of a wide variety of diseases are well known, especially for their use in cancer treatment [55]. Due to the limitations of the mAbs’ antitumor efficacy, attempts are being made to improve the potential efficacy of mAbs. These efforts encompass the conjugation of mAbs to radionuclides, fusion with immunotoxins, or coupling to ADCs. The coupling of a mAb with a cytotoxic agent or a small molecule is called payload [56].

An ADC consists of 3 crucial elements: (a) a monoclonal antibody, (b) a chemical linker, and (c) an anti-neoplasm payload [57]. Currently there is a sustained challenge to optimize ADCs, with the principal focus of R&D aimed at the mAb or the chemical linker, concurrent with small-scale attempts directed at the optimization of cytotoxic payloads. Among the 114 completed or ongoing human trials, there is a lack of diversification in the medicinal payloads utilized, with only 7 payload preparations reported, (4 additional trials are ongoing with non-reported structures). Six of seven payload mixtures are derived from natural product sources, highlighting the value of natural products as cytotoxic payloads for ADC in research studies [58].

Auristatins and their relatives (MMAE and MMAF) derived from marine cytotoxins maintain a valuable function in ADC payload technology. This composite methodology has an invaluable influence on the pharmacokinetics of cytotoxic preparations and can extend the half-life of these drugs from hours to days [6,8].

Based on a recent 1983 to 2002 FDA review, from 79 anticancer and antiviral approved medications, 9 cytotoxins were obtained from plants and 21 were natural product preparations [59]. Additionally, from 39 anticancer molecules, 13 of them were based on natural products. Sixty percent of current medicinal products are bioengineered based on natural origin [59].

## 5. Drug-Antibody Ratio (DAR)

The drug–antibody ratio (DAR), or number of drug molecules conjugated to a single ADC, is very important for the determination of efficacy of ADCs. DAR widely varies and depends on other ADC variables [60]. The DAR values are also dependent on the site of conjugation and the use of light or heavy conjugated chains [61]. The DAR value influences the effectiveness of the medicine due to the depression in potency caused by low drug loading, while elevated drug loading can impact toxicity and pharmacokinetics (PK) [62].

## 6. Glycosylation

Many proteins for biological activity require glycosylation [63]. Glycosylation is the addition of a carbohydrate to an organic molecule such as protein. Modifying glycosylation via numerous engineering techniques has contributed to advances in immunotherapies, such as antibodies, cell-based therapies, and recombinant proteins [64]. The impact of glycosylation on ADCs has not been fully well evaluated and further investigation is needed [65].

## 7. Pegylation

Polyethylene glycol (PEG) is manufactured by the interaction of ethylene oxide with water, ethylene glycol, or ethylene glycol oligomers, catalyzed by acidic or basic catalysts [66]. Attachment of PEG to a protein is called pegylation. PEG elements vary in geometry and molecular weight. Pegylation enhances the protein’s medicinal effect by modifying its pharmacokinetics [67]. Pegylation has three main advantages: (a) improving pharmacokinetics and pharmacodynamic properties, (b) enhancing drug stability, and (c) improving distribution of the drug in the body [68]. Pegylation on ADCs as a linker may be helpful in improving the solubility and reducing the aggregation of ADCs [69]. This indicates that pegylation is useful in enhancing the PK and therapeutic efficiency of ADCs [70].

## 8. Charge

The charge of a protein is an important factor which can influence the PK of a protein. The isoelectric point is between 8 and 9, at which the protein conveys no net electrical charge [71]. It is theorized by some researchers that the nascent peptide charge may impact translation, a factor regulating protein expression and translation efficiency. At the moment, there is not much information regarding the impact of charge on the ADCs. However, it should be considered during the ADC development process [72].

## 9. Pharmacokinetics

Pharmacokinetics (PK) is the discipline depicting the timeline of medication absorption, distribution, metabolism, and excretion [73]. Pharmacokinetics is a quantitative assessment of how living systems deal with xenobiotics [74]. Over the years, pharmacokinetics has become a very important tool in drug development [75]. Pharmacokinetics can also be used in clinical settings as it can provide a starting point for dose selection involving a patient population, or in personalized medicine [76]. A detailed description of the pharmacokinetics of ADCs is beyond the scope of this review. A current review on the clinical pharmacology of ADCs highlights the importance of pharmacokinetics and pharmacodynamics in the development of ADCs [11].

## 10. Preclinical Studies of ADCs

Preclinical studies are conducted to determine a starting dose for human study and assess the toxicity of the product. The preclinical evaluation of ADCs should involve antibody/antigen binding studies, in-vitro cytotoxic measurements, in-vivo anti-tumor efficacy analysis, pharmacokinetics, and toxicology research in rodent and non-human primates [76].

At the preclinical stage, it is important to comprehend the pharmacokinetics of an ADC in combination with its in-vitro and in-vivo activity, obtaining knowledge of its methods of operation in order to optimize and select the proper ADC for the best efficacy and safety studies. In defining the PK of an ADC correctly, components of ADCs like the total antibody, conjugated and unconjugated cytotoxin, DAR distribution, and metabolites in various elements such as bile, plasma, and tissues from in vitro or in vivo studies are necessary [77]. In a research study, the authors recommended the following studies that can be performed preclinically to define the PK of ADCs [78]:

“I. In vitro stability studies in plasma from different species to understand linker stability as well as mechanisms of deconjugation across species.

II. In vitro catabolism studies to determine the types of catabolites/metabolites formed and whether they have any activity within in vitro cell potency assays.

III. In vivo PK and exposure of the various analytes in the efficacy and toxicity species to characterize the PK, determine PK drivers of efficacy/toxicity, establish in vitro–in vivo correlations of stability and mechanisms of deconjugation/catabolism.

IV. Biodistribution studies to look for tumor and normal tissue uptake (specific or non-specific), and in vivo catabolite profiles in various tissues, including understanding any contribution of catabolites to any bystander effects.

V. In vitro potency, CYP, and transporter profiling of the cytotoxic drug to evaluate the risk of possible drug-drug interactions in the clinic.

VI. Utilize in vivo exposure data at the efficacious and toxic doses to estimate therapeutic index.

VII. Prediction of human PK to estimate efficacious dose and schedule in patients.”

## 11. Adverse Effects of ADCs

Adverse effects differ depending on the class of targeted antibody and what it actually targets, and can be affected by its locus and class of cancer as well as a patient’s all-around fitness. Proteins produced by the body and targeted by antibodies are expressed by diseased and healthy cells. Collateral damage my occur result via targeted antibodies going off-target, yielding immune responses and resulting in adverse effects [79].

Adverse effects can vary from life-threatening to moderate or to mild, depending upon various conditions. In the majority of patients, adverse effects can be easily managed once they are addressed early [80].

Standard adverse reactions affiliated with antibody therapy can include diarrhea, fatigue, pruritus, constipation, hemorrhage, thrombocytopenia, peripheral neuropathy, pain, infusion reactions, infection, nasopharyngitis, anemia, appetite loss, nausea, cough, fever, pneumonia, headache, rash, hypokalemia, lymphopenia, neutropenia, pyrexia, and vomiting [81].

## 12. Anticipated Directives of Antibody Drug Conjugates

Numerous cytotoxic medications have been acknowledged involving amatoxins, microtubule inhibitors, and anthracyclines, which are significant extensions of maytansinoids and auristatins. New linkers have been developed improving the therapeutic effects of ADCs [81]. Combination strategies currently explored in numerous clinical trials include combinations with checkpoint PD-1 or PD-L1 inhibitors or traditional chemotherapies. The evolution of innovative ADCs offers substantial advancements for prospective neoplasm therapy [82]. Researchers have proposed the following stringent criteria to address before an ADC moves between initial clinical trials into prospective clinical development:

(1) Patients preferred for target neoplasm antigen expression, satisfactory reactions recorded in those individuals involving gene amplification or high expression.
(2) Dosage-limited off-mark cytotoxicity should be less than what is anticipated from medicinal payload.
(3) Verify the medical preparation payload is applicable for the indications for use.

ADCs developed may also be applied to other antibody-related therapeutics, including bi-specific antibodies, antibodies, or antibody fragments fused with a toxin (immunotoxins) with new avenues utilizing naturally derived cytotoxins, chimeric antigen receptors, and radiolabeled antibodies (radioimmunoconjugates) [83].

## 13. Conclusions

This review summarizes the progress and obstacles of ADCs, with emphasis on antibody conjugation, linker-payload chemistry, innovative payload categories, pharmacokinetics, and production development.

The limitations and failures of various ADCs have been linked to unresolved efficacy and toxicity due to low drug internalization rates, low levels of target antigen expression and off-target toxicities due to inadvertent drug release, leading to the research of new methodologies including: (a) bispecific ADCs, and (b) combinations of ADCs and immunotherapy. It is anticipated that, in the future, more safe and efficacious ADCs will be developed, and these ADCs can also be used for other diseases beyond cancer.

## Figures and Tables

**Table 1:** FDA approved ADCs and their characteristics.

Trade Name	Generic Name	Conjugate	Indication	Target	Year of Approval
MYLOTARG	Gemtuzumab ozogamicin	Calicheamicin	Hematological	CD33	2010/2017
ADCETRIS	Brentuximab vedotin	Monomethyl auristatin E (MMAE)	Hematological	CD30	2011
BESPONSA	Inotuzumab ozogamicin	Calicheamicin	Hematological	CD22	2017
POLIVY	Polatuzumab vedotin	Monomethyl auristatin E (MMAE)	Hematological	CD79b	2019
KADCYLA	Trastuzumab emtansine	Myatansinoid (DM1)	Solid tumor	HER2	2013
ENHERTU	Trastuzumab deruxtecan	Deruxtecan (Dxd)	Solid tumor	HER2	2019
PADCEV	Enfortumab vedotin	Monomethyl auristatin E (MMAE)	Solid tumor	Nectin-4	2019
TRODELVY	Sacituzumab govitecan	Govitecan SN-38	Solid tumor	Trop-2	2020
BLENREP	Belantamab mafodotin	Microtubule inhibitor MMAF	Myeloma	BCMA	2020
ZYNLONTA	Loncastuximab tesirine-lpyl	SG3199	B-cell lymphoma	CD19	2021

Taken and modified from Mahmood, I. Clinical Pharmacology of Antibody-Drug Conjugates. Antibodies. 2021; 10: 20. [11].

**Table 2:** ADC linker technology and the release mechanism.

ADC Linker Technology	Release Mechanism
Disulfide	Designed to be cleaved through disulfide exchange with an intracellular thiol, such as glutathione.
Hydrazone	Designed for serum stability and degradation in acidic compartments within the cytoplasm.
Peptide	Designed to be enzymatically hydrolyzed by lysosomal proteases such as cathepsin *B*.
Theoeither	Nonreducible and designed for intracellular proteolytic degradation.

## Data Availability

Data available from references provided in manuscript.
